# A transdisciplinary systems-level approach to maternal health: Developing a Research Center of Excellence

**DOI:** 10.1017/cts.2026.10778

**Published:** 2026-07-06

**Authors:** Anne Elizabeth Glassgow, Sage J. Kim, Amanda Knepper, Zeynep Madak-Erdogan, Arden Handler, Tamara Hamlish, Jess Rothstein, Aiman Soliman, Rachel Caskey

**Affiliations:** 1 University of Illinois Chicago College of Medicine, Chicago, IL, USA; 2 School of Public Health, University of Illinois Chicago, Chicago, IL, USA; 3 University of Illinois Urbana-Champaign, Champaign, IL, USA

**Keywords:** maternal health, transdisciplinary research, community-academic partnerships, research career development, research center of excellence

## Abstract

The United States faces a critical maternal health crisis, characterized by the highest mortality rates among high-income nations and profound racial disparities. The University of Illinois Chicago established the Luma Center, a Maternal Health Research Center of Excellence funded by the National Institutes of Health IMPROVE initiative. This article details the center’s development, implementation, and theoretical foundation. Central to the Luma Center’s mission is the Biopsychosocial Ecosystem Framework, which shifts the research focus from individual-level factors to the multilevel systemic drivers of maternal morbidity and mortality. Utilizing a transdisciplinary approach involving 18 academic disciplines, the Center operates through five core areas: Leadership, Community Partnership, Research, Training, and Data. The primary objectives are to conduct robust multilevel research, cultivate a diverse research workforce, and strengthen community partnerships to ensure the translation of findings into effective postpartum interventions. By integrating community expertise with academic innovation, the Luma Center seeks to systematically address the fundamental causes of poor maternal health outcomes and advance science to promote perinatal health and wellness.

## Introduction

The United States (U.S.) continues to have the highest maternal mortality rate among high-income nations. Maternal mortality rates reveal persistent differences by race [1]. In 2023, Black women had the highest maternal mortality rate at 50.3 deaths per 100,000 live births compared with White (14.5 per 100,000), Hispanic (12.4 per 100,000), and Asian (10.7 per 100,000) women [2]. Maternal Mortality Review Committees estimate that more than 80% of pregnancy-related deaths are preventable [1,3–5]. This preventability is complex and influenced by a constellation of patient, provider, facility, and community factors [6–8].

Addressing the national maternal health crisis requires innovative, multilevel research approaches that capture the multifaceted nature and intricate interplay between the drivers that contribute to poor maternal health outcomes. The synergized efforts of perinatal health researchers, academic and health institutions, and community organizations are imperative to both uncovering and systematically addressing fundamental causes of maternal morbidity and mortality. To this end, transdisciplinary research and dissemination efforts, undertaken with active community partner collaborations, are essential to translating research findings into novel interventions that address the complex, integrated systems that contribute to disparate maternal health outcomes.

This article describes the establishment and operational structure of the University of Illinois Chicago (UIC) Maternal Health Research Center of Excellence, titled the “Luma Center” that supports interprofessional research, workforce development, and community collaboration toward the dissemination of interventions for improving maternal health care. The Luma Center provides a compelling proof of concept, transitioning beyond theoretical toward a concrete, scalable blueprint for integrated, transdisciplinary research. It operationalizes inclusive excellence by shifting the focus from simply identifying barriers to demonstrating the infrastructure required to dismantle them through intentional workforce development and community collaboration. The Luma Center transcends traditional outreach by establishing community partnerships that institutionalize the integration of living experience directly into every phase of the research lifecycle, from initial design to final dissemination. The Luma Center serves as a paradigm for transdisciplinary synergy, demonstrating how the deliberate convergence of research, clinical training, and community partnerships within a single conceptual framework fosters a more responsive and dynamic scientific ecosystem capable of accelerating the translation of research findings into community-based clinical practice. A contextual and conceptual overview of the Luma Center is provided along with a description of the guiding premises, which focuses on understanding and addressing the primary drivers of poor maternal health to develop and implement the most appropriate and effective intervention strategies. The Luma Center’s core foci are described in terms of their objectives, activities, and expected outcomes. Finally, directions for future research, training, and community integration and dissemination are presented.

## Methods

### Background

In 2019, to address the high rates of maternal morbidity and mortality and promote maternal health in the U.S., the National Institutes of Health (NIH) funded the Implementing a Maternal Health and Pregnancy Outcomes Vision for Everyone initiative [9]. To promote novel research toward the enhancement of the health outcomes of pregnant and postpartum women who experience the direst health outcomes, NIH issued a funding opportunity announcement in August 2022 for Maternal Health Research Centers of Excellence as part of the Implementing a Maternal Health and Pregnancy Outcomes Vision for Everyone initiative. In June 2024, the Luma Center was one of the funded Centers of Excellence.

**Figure 1.**
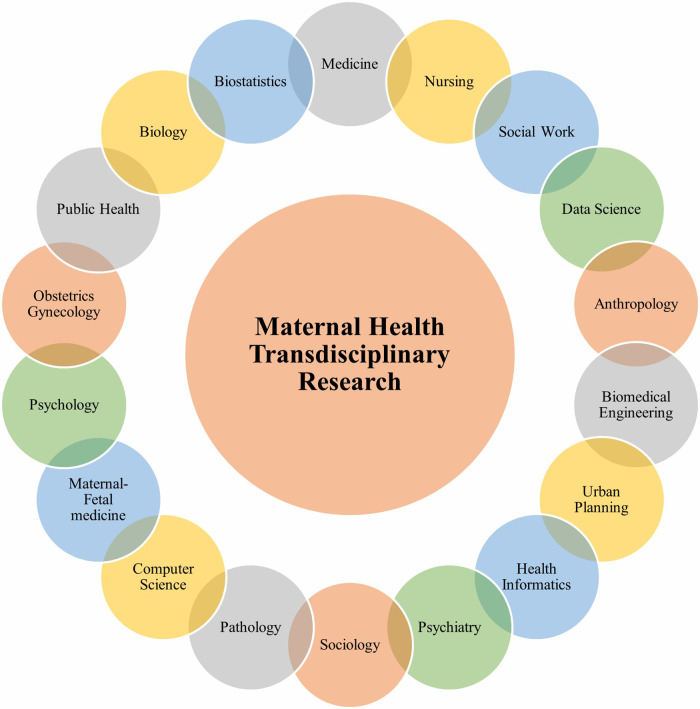
Figure 1 long description. Academic disciplines represented on the Luma Center transdisciplinary research team.

Maternal health research is complex and requires a scientific approach that integrates the study of the interaction between the biological, behavioral, socioecological, and clinical factors that influence maternal health outcomes. To address this complexity, a transdisciplinary team of maternal health researchers with complementary experience from across UIC was convened to respond to the funding opportunity. The team integrated knowledge from their 18 different academic disciplines to create innovative conceptual, theoretical, methodological, and translational innovations that move beyond discipline-specific approaches to address maternal health (Figure 1).

The overall goal of the Luma Center is to advance the science of maternal health by examining the ways in which fundamental factors, such as economic conditions and the built environment, contribute to severe maternal morbidity and mortality [10,11]. The four primary aims of the Luma Center are to (1) conduct robust transdisciplinary maternal health research, using a multilevel approach; (2) serve as a hub for career development and workforce development for maternal health research; (3) disseminate effective strategies for improving comprehensive postpartum care; and (4) facilitate and strengthen community partnerships to support the development of, and engagement in maternal health research.

### Setting and context

The UIC is a highly respected research-intensive university. Situated in the heart of Chicago, with a long-standing mission of social justice, civic engagement, and the promotion of the health of underserved populations, UIC is well-respected among its peer institutions for its research innovation and commitment. Chicago is the third largest city in the U.S. and is home to over 2.7 million residents living in 77 community areas. Both in Chicago and across the state of Illinois, rates of severe maternal morbidity and maternal mortality are higher than the national average [3].

### Framework

Maternal health outcomes cannot be understood solely at the individual level; research must shift towards models and approaches that measure fundamental, multi-level drivers. Indeed, the impact of upstream systemic drivers is the underpinning of disease and has been well-documented for a wide range of health outcomes [12–15]. Nevertheless, very few studies incorporate specific measures and use data to link systems-level factors with adverse maternal health outcomes. Conceptually, the biopsychosocial ecosystem as a contributing mechanism to adverse maternal health is under-investigated, and there are theoretical and methodological gaps that have yet to be addressed [16,17].

**Figure 2.**
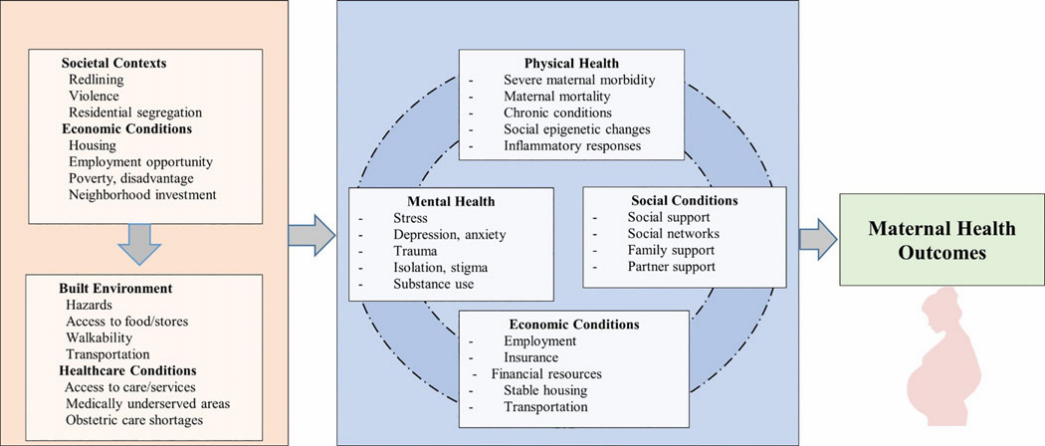
The Luma Center Biopsychosocial Ecosystem Framework for maternal health research.

The thematic focus of the Luma Center is to synergize the study of multilevel systems of change for maternal health. Figure 2 displays our theoretical Biopsychosocial Ecosystem Framework for maternal health research which details how contextual factors (i.e., societal contexts, economic conditions, built environment, and healthcare conditions) are systemic, fundamental factors that influence and contribute to multiple aspects of maternal health and wellbeing outcomes (i.e., physical health, mental health, economic conditions, and social conditions). The Biopsychosocial Ecosystem Framework synthesizes the Socioecological Model [18] and the Social Determinants of Health framework [19] to map how multi-level systemic drivers directly influence physiological maternal outcomes. By further incorporating the Life Course Model [20], our framework accounts for the longitudinal accumulation of risk and the weathering effects of communicative upstream inequities and stress over time. This integrated approach addresses existing theoretical gaps by providing a structured methodology to link macro-level systemic factors with individual biological stressors. The main premise of the Biopsychosocial Ecosystem Framework is that the etiology of maternal health outcomes originate from systemic societal and economic conditions that limit access to adequate socioeconomic resources and increase risk of exposure to harmful built and non-built environments [21]. Women at risk for severe maternal morbidity and mortality are profoundly affected by systemic, upstream factors – such as adverse built and non-built environments and lack of access to essential care and services – which induce chronic physical and psychological stress, ultimately contributing to poor maternal health outcomes.

## Results

The LUMA Center established four specialized Cores, Leadership, Research, Community Partnership, and Training, alongside a Data Nexus to execute the activities required to achieve its primary aims: (1) conduct robust transdisciplinary maternal health research, using a multilevel approach; (2) serve as a hub for career development and workforce development for maternal health research; (3) disseminate effective strategies for improving comprehensive postpartum care; and (4) facilitate and strengthen community partnerships to support the development of, and engagement in maternal health research. The Leadership Core provides the strategic governance and rigorous oversight necessary to ensure the Luma Center’s four primary aims are met with both scientific precision and operational excellence. The Research, Training, and Community Partnership Cores (CPC) lead Aims 1, 2, and 4, respectively, whereas all cores collaborate to address Aim 3. While the Cores collectively advance the Luma Center’s overarching aims, each maintains distinct, core-specific aims or goals.

### Leadership Core

**Figure 3.**
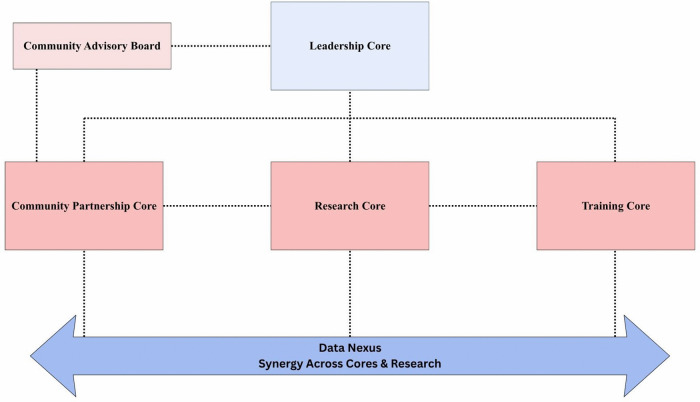
The Luma Center organizational structure.

**Figure 4.**
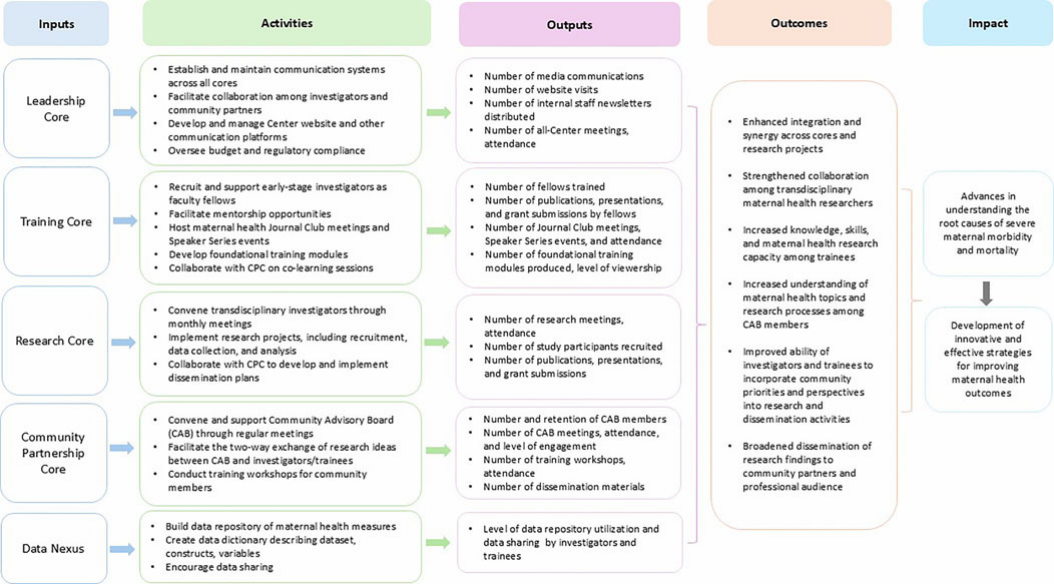
Figure 4 long description. The Luma Center logic model.

The Luma Center’s organizational structure (Figure 3) assembles researchers, leaders, community partners, and mentors with collective expertise to advance maternal health research, training, and community partnerships. The organizational structure provides synergy across the Cores. Figure 4 describes the Center Logic Model that includes the cores (inputs), activities, outputs, outcomes, and impact. Led by the Luma Center Co-Principal Investigators, the Leadership Core oversees research direction, integration across research project and cores, cultivation of strong partnerships, and support for operations.

### Research Core

The Research Core directs the Luma Center Aim 1 to conduct robust transdisciplinary maternal health research, using a multilevel approach. The Research Core convenes transdisciplinary investigators whose complementary expertise provides scientific leadership and collaboration across all Luma Center research, cores, and activities. The Research Core comprises Center investigators, staff, and trainees. The goals are to: (1) mobilize investigators for a unified research vision; (2) support investigators and trainees (e.g., advising on innovative research approaches, addressing current conceptual issues and methodological limitations); (3) advance research progress and dissemination (e.g., providing access to data, reconciling core measures, and offering research guidance and technical support); and (4) ensure that research is conducted with scientific integrity. The research vision is to support research that examines multi-level (individual, interpersonal, community, environmental, health system, societal) systems to improve research methodology and maternal health outcomes.

To ensure the realization of its core vision and goals, the Research Core facilitates monthly transdisciplinary team meetings that serve as a forum for the systematic review of multi-source data, the collaborative resolution of emerging methodological challenges, and the strategic planning of scholarship and dissemination activities. The Luma Center investigators, who represent 18 academic disciplines (Figure 1), are part of the Research Core and are invited to attend the meetings. These meetings provide a critical venue for vetting emerging research concepts and preliminary findings while simultaneously fostering cross core partnerships that also integrate the Training Core and the CPC into the Luma Center research infrastructure. By involving fellows, trainees, and the CPC, these sessions serve as an incubator for innovative research and provide a structured environment for mentorship, allowing for the collective interrogation of preliminary data and iterative troubleshooting.

To uphold the highest standards of integrity and regulatory compliance, the Research Core established a comprehensive Policy and Procedures Manual. This foundational document provides mandatory guidance for all Luma Center affiliates including faculty, staff, and trainees, ensuring that every phase of the research lifecycle adheres to ethical guidelines and rigorous data management. This framework ensures that all scholarly products including peer reviewed publications, conference presentations, and community facing reports are accurate, high quality, and scientifically valid, ultimately prioritizing the timely translation of data into actionable knowledge.

The Luma Center’s primary research project titled, “Multilevel exposure to adversity across the life-course: Quantifying biological implications in urban postpartum women,” is a five-year, prospective, explanatory sequential-mixed-methods cohort study. The aims are to 1) describe the exposure to multilevel (neighborhood, family, and individual) adverse risk factors among urban postpartum women; 2) assess the effect of exposures on bio-physical outcomes (multi-omics profiling of peripheral blood mononuclear cells, including DNA methylation, chromatin accessibility, and histone modification alongside inflammatory and steroid hormone assays); and 3) understand the context of daily experience across the life-course of postpartum women using qualitative photovoice methods. This study integrates interviewer-administered survey data on individual and family characteristics with longitudinal electronic health records and geocoded neighborhood-level information. Findings from this study can advance understanding of the biological embedding of adversity and develop new knowledge about the impact of social-environmental conditions on women’s living experiences.

### Data Nexus

The Data Nexus is the conduit for synergy across cores, which aims to coordinate research. The primary goal of the Data Nexus is to construct a Data Repository of common maternal health measures that are continuously expanded to stimulate and facilitate research. Compiling common data elements is crucial for comparing groups, areas, and temporal trends across research projects within the Center and potentially across other funded Maternal Health Research Centers of Excellence, thereby advancing maternal health science. The objectives of building a Data Repository are to: (1) catalog and disseminate key maternal health measures; (2) provide theoretical and analytic frameworks to guide research activities; and (3) encourage investigators to use comparable maternal health data. The Data Repository includes validated surveys, electronic health records, biological indicators (i.e., blood-inflammatory markers, epigenetic markers, biomarkers, and urine-environmental toxins), qualitative interview transcripts, and population-level measures. Data Nexus provides the structure for oversight of the Center’s accurate and timely collection, management, analysis, and secure data sharing. The Data Nexus supports the development of maternal health theory, analytic methods, and translational science by standardizing data elements and maximizing data sharing.

### Training Core

The Training Core oversees the Luma Center Aim 2 to serve as a hub for career and workforce development for maternal health research. The specific aims of the Training Core are to: 1) increase the maternal health research pipeline by recruiting and supporting the academic career development of postdoctoral and junior faculty fellows from a variety of clinical and public health backgrounds; 2) develop and successfully implement a robust training program for early-stage investigators, including junior clinical and public health faculty and postdoctoral fellows, to enable them to address a variety of research questions related to improving maternal health outcomes using innovative quantitative and qualitative research methods and strategies; 3) provide community-based clinical partners with a robust research training experience that will enable them to successfully undertake or partner with investigators to conduct maternal health research; and 4) provide a strong research and journey-mentoring program to early-stage investigators to enable them to effectively address maternal health issues including improving maternal health outcomes.

The Training Core provides funding and mentoring support to early-career fellows and trainees from diverse academic and clinical disciplines – including psychology, pathology, biology, medicine, nursing, and public health – who are affiliated with UIC (e.g., postdoctoral fellows, junior faculty) or the community (e.g., healthcare providers). The Core leverages established training programs, research activities, and community partnerships to develop trainees committed to maternal health research. Trainees explore biological, behavioral, environmental, sociocultural, and structural factors impacting pregnancy-related mortality and severe maternal morbidity.

The Luma Center fellows and trainees are recruited from five distinct groups within the UIC College of Medicine and School of Public Health:

- Group 1 comprises scholars from the Building Interdisciplinary Research Careers in Women’s Health (BIRCWH) Program, an NIH-funded K12 grant [22]. The Luma Center supports one additional junior faculty BIRCWH Associate annually whose research focuses on maternal health.
- Group 2 is drawn from the Precision Lifestyle Medicine and Translation Research (PREMIER) Postdoctoral Training Program (NIH T32) [23]. PREMIER is an interprofessional program that focuses on precision, proactive, and personalized care for chronic conditions; the Luma Center provides research support to one maternal health-focused postdoctoral fellow from this cohort each year.
- Group 3 consists of participants from the Academic Career Enhancement Program for Maternal and Child Health (ACE-MCH) at the School of Public Health Center of Excellence in Maternal and Child Health [24]. The Luma Center provides research support for one to two postdoctoral fellow annually from this pipeline.
- Group 4 includes clinicians or staff from AllianceChicago, a national network of community health centers [25]. Through this partnership, the Luma Center funds one fellow per year to receive specialized training in maternal health research.
- Group 5 includes Luma Center early-stage investigators, postdoctoral fellows, and research assistants. While these individuals are not required to follow a specific curriculum, they have full access to all Luma Center training opportunities and resources.

Fellows from Groups 1–4 are primarily recruited through established training and clinical programs (BIRCWH, T32, ACE-MCH, and AllianceChicago). For Group 5, the Center identifies trainees by leveraging UIC’s extensive internal networks, including departmental listservs, clinical training hubs, and collaborative academic forums. In collaboration with the clinical and training programs, the Luma Center interviews and selects fellows who align with its maternal-health research goals. As of March 2026, from Groups 1–4, the Luma Center has one BIRCWH, one T32, two ACE-MCH, and one AllianceChicago fellows. From Group 5, the Luma Center has three postdoctoral fellows and eight trainees who are research assistants.

Once selected, participants are matched with a Luma Center research mentor who supports their professional development throughout the fellowship. Each fellow completes a Research Development Plan to outline individualized training needs and performs an annual self-assessment to monitor progress. To support the execution of their work, the Luma Center awards funds for projects addressing maternal health research. Potential research areas span epidemiology, data science, community-engaged research, implementation science, and policy- or systems-level research. Mentors provide ongoing guidance as fellows identify opportunities, refine study aims, and gain hands-on experience in various methodologies. Fellows participate in multidisciplinary curricular and mentoring opportunities including, foundational training and a monthly Journal Club, designed to enhance their scientific knowledge and prepare them to conduct both quantitative and qualitative maternal health research.

The Training Core collaborates with the CPC to offer fellows opportunities to engage with the Community Advisory Board (CAB) via bimonthly co-learning sessions. These iterative, dialogue-based sessions foster bidirectional exchange of knowledge between researchers and community stakeholders. This collaboration deepens fellows’ understanding of community-engaged research that is truly responsive to community needs.

The Training Core also is developing foundational online training modules covering key maternal health topics to build the knowledge and skills of Luma Center fellows and trainees as well as the broader maternal health workforce. These modules complement other Luma Center wide learning opportunities, including the monthly Journal Club and quarterly Speaker Series, both of which are open to the Luma Center affiliated staff and faculty as well as external partners. The Speaker Series disseminates timely updates on maternal health research, clinical practice, and emerging policy issues to a broad audience of practitioners, researchers, and community-based organizations. Brief day of surveys and an annual Training Core evaluation survey assess participant satisfaction and measure the impact of these offerings on participants’ knowledge, research practice, and collaborations.

### Community Partnership Core and Community Advisory Board

The CPC is responsible for Aim 4 to facilitate and strengthen community partnerships to support the development of, and engagement in maternal health research. The CPC coordinates collaborative efforts among community members affected by maternal morbidity and mortality and researchers to enhance research outcomes. The goals of the CPC are to facilitate, coordinate, and evaluate community engagement and dissemination across all research within the Luma Center. The CPC recognizes the unique expertise of all partners, including community members and community organizations, healthcare providers, policymakers, and researchers, to create an environment where all voices are heard and all partners contribute to research. CPC meetings occur at a monthly cadence, and allow for a dynamic exchange of research updates, ideas, and emerging findings across the Luma Center.

The CPC partners with two community-based organizations, EverThrive Illinois and AllianceChicago. EverThrive Illinois is a non-profit organization focused on achieving reproductive justice and health equity for all people and families in Illinois. Their expertise is convening and mobilizing community members, health care providers, social service providers, and elected officials to work toward the advancement of optimal maternal health [26]. AllianceChicago, a Health Center Controlled Network of 200 community health centers across the U.S., contributes to the CPC as a healthcare partner. AllianceChicago provides a platform to engage with community-based clinicians and clinics to increase knowledge and inform translation of research into settings outside of academic health care [25].

The CPC is guided by the bidirectional aim of bringing research to the community and bringing community to the researcher. The team collaborates with the Training Core on the development and delivery of training workshops, whereby community members can be introduced to the basic principles of scientific research, become familiar with community-level maternal health data, and develop strategies for community members to collaborate with researchers to disseminate research.

The CPC Core and EverThive Illinois co-lead the CAB which is composed of 10 community members from across the state who represent women, families, patients, and community groups. The CAB is an equitable partner that brings community experience and voice to the Center’s research and training activities. CAB meetings are held at least six times per year and provide a platform for CAB members to engage with researchers. As part of the Center evaluation process, feedback is collected from CAB members following each meeting, and annual interviews are conducted with each member to explore their experiences, perceived benefits of participation, and suggestions for enhancing collaboration. In addition, interviews are conducted with researchers who engage with the CAB to understand how they integrate community input in their research and dissemination efforts.

The Luma Center’s anchor research project is titled: “Multilevel exposure to adversity across the life-course: Quantifying biological implications in urban postpartum women.” This project investigates the impact of social epigenetics on maternal health outcomes, specifically examining how systemic root causes create unequal exposure to adversity that influences biophysical changes in postpartum women. This mixed-methods study employs a prospective, cross-sectional design, collecting data from multiple sources, including community-level data, patient surveys, clinical data, biological data, and qualitative interviews with postpartum women. The goal of this project is to illuminate how social and environmental factors create unequal exposure to adversity and impact biophysical health and mental health outcomes in postpartum women.

## Discussion

The Luma Center is an innovative Maternal Health Research Center committed to the creation of synergy between researchers and the community to advance science and develop a highly trained workforce committed to improving maternal health. Our framework, which posits structural root causes as the fundamental drivers of adverse maternal health outcomes, has been instrumental in guiding our research, community partnerships, and training initiatives. To disrupt the effects of systemic mechanisms, this multilevel approach focuses on the development of new theoretical approaches, improved data analytics, the identification of multilevel mechanisms that contribute to poor health, and the strengthening of maternal care by addressing its multiple dimensions (i.e., physical, behavioral, social, and economic) to improve health and wellbeing in pregnant and postpartum women. The unique focus is advantageous, and we are well-positioned to address the profound theoretical and methodological gaps that currently exist in this area of science.

Beyond the framework, the Luma Center’s distinctiveness is rooted in its convergence of 18 diverse academic disciplines, fostering a transdisciplinary environment essential for addressing the multifaceted complexities of maternal health science. Situated in Chicago, the Luma Center is strategically positioned to investigate urban maternal health and disparate outcomes prevalent in major metropolitan areas [27,28]. Our research specifically prioritizes the postpartum period, a critical clinical window characterized by the highest prevalence of maternal mortality [29,30]. By concentrating on this sensitive timeframe within a high-density urban context, the Luma Center is uniquely equipped to dismantle the systemic barriers that drive inequitable maternal health trajectories.

This article describes the Luma Center’s design and operational structure. As such, an evaluation of its impact on maternal health research has not yet been conducted. However, the robust foundation, which includes a transdisciplinary team, strategic cores, cohesive community partnerships, and an innovative data nexus, positions it to significantly advance the field. A longitudinal formal evaluation of the Luma Center’s effectiveness and overall contribution to science is underway.

## Conclusion

The Luma Center is investigating the mechanisms by which systemic root causes affect maternal health and how interventions that mitigate the effects of adverse factors and conditions at multiple levels provide an opportunity for the restoration of holistic maternal health and wellbeing. When individual-level health interventions are paired with community-level efforts to increase the capacity necessary to overcome health access barriers, maternal health is promoted. The Luma Center aims to generate new knowledge, strengthen the maternal health workforce, and ultimately create a healthier future for all women and families. Finally, sustained federal funding is essential for advancing maternal health research, cultivating a dedicated workforce of researchers, and effectively translating scientific findings into measurable improvements in maternal health outcomes nationwide.

## Figure 1. Long description

A Venn diagram representing the academic disciplines involved in the Luma Center transdisciplinary research team. The diagram consists of 15 overlapping circles, each representing a different academic discipline. The central circle, labeled Maternal Health Transdisciplinary Research, is the intersection of all the disciplines. The surrounding circles are labeled as follows: Biology, Biostatistics, Medicine, Nursing, Social Work, Data Science, Anthropology, Biomedical Engineering, Urban Planning, Health Informatics, Psychiatry, Sociology, Pathology, Computer Science, Maternal-Fetal Medicine, Psychology, Obstetrics Gynecology, and Public Health. Each circle overlaps with several others, indicating the interdisciplinary nature of the research team. The overlaps signify the collaboration and integration of knowledge from different fields to address maternal health issues.

Navigate back to Figure 1..

## Figure 4. Long description

A diagram of the Luma Center’s organizational structure, showing the inputs, activities, outputs, outcomes, and impact across different cores. The structure includes five main cores: Leadership Core, Training Core, Research Core, Community Partnership Core, and Data Nexus. Each core has specific activities that lead to measurable outputs and outcomes, ultimately impacting maternal health research and outcomes. The Leadership Core focuses on communication and collaboration, the Training Core on educating early-stage investigators, the Research Core on conducting research projects, the Community Partnership Core on engaging community members, and the Data Nexus on building data repositories. Arrows indicate the flow from inputs through activities to outputs, outcomes, and finally impact.

Navigate back to Figure 4..
